# Effects of Alda-1, an Aldehyde Dehydrogenase-2 Agonist, on Hypoglycemic Neuronal Death

**DOI:** 10.1371/journal.pone.0128844

**Published:** 2015-06-17

**Authors:** Tetsuhiko Ikeda, Tetsuya Takahashi, Mika Tsujita, Masato Kanazawa, Masafumi Toriyabe, Misaki Koyama, Kosuke Itoh, Tsutomu Nakada, Masatoyo Nishizawa, Takayoshi Shimohata

**Affiliations:** 1 Department of Neurology, Brain Research Institute, Niigata University, Niigata, Japan; 2 Department of Center for Integrated Human Brain Science, Brain Research Institute, Niigata University, Niigata, Japan; Stellenbosch University, SOUTH AFRICA

## Abstract

Hypoglycemic encephalopathy (HE) is caused by a lack of glucose availability to neuronal cells, and no neuroprotective drugs have been developed as yet. Studies on the pathogenesis of HE and the development of new neuroprotective drugs have been conducted using animal models such as the hypoglycemic coma model and non-coma hypoglycemia model. However, both models have inherent problems, and establishment of animal models that mimic clinical situations is desirable. In this study, we first developed a short-term hypoglycemic coma model in which rats could be maintained in an isoelectric electroencephalogram (EEG) state for 2 min and subsequent hyperglycemia without requiring anti-seizure drugs and an artificial ventilation. This condition caused the production of 4-hydroxy-2-nonenal (4-HNE), a cytotoxic aldehyde, in neurons of the hippocampus and cerebral cortex, and a marked increase in neuronal death as evaluated by Fluoro-Jade B (FJB) staining. We also investigated whether *N*-(1,3-benzodioxole-5-ylmethyl)-2,6-dichlorobenzamide (Alda-1), a small-molecule agonist of aldehyde dehydrogenase-2, could attenuate 4-HNE levels and reduce hypoglycemic neuronal death. After confirming that EEG recordings remained isoelectric for 2 min, Alda-1 (8.5 mg/kg) or vehicle (dimethyl sulfoxide; DMSO) was administered intravenously with glucose to maintain a blood glucose level of 250 to 270 mg/dL. Fewer 4-HNE and FJB-positive cells were observed in the cerebral cortex of Alda-1-treated rats than in DMSO-treated rats 24 h after glucose administration (*P* = 0.002 and *P* = 0.020). Thus, activation of the ALDH2 pathway could be a molecular target for HE treatment, and Alda-1 is a potentially neuroprotective agent that exerts a beneficial effect on neurons when intravenously administered simultaneously with glucose.

## Introduction

Transient hypoglycemic episodes occur during treatment of diabetes mellitus with insulin or oral hypoglycemic drugs and in cases involving insulinoma, alcoholism, anorexia nervosa, or others. Although many patients recover with appropriate treatment, transient hypoglycemic episodes can result in coma, seizures, and myriad other global and focal neurological deficits [1–3]. In an analogy to hypoxic encephalopathy, this syndrome has been termed hypoglycemic encephalopathy (HE). The only treatment for HE is blood glucose (BG) correction by glucose administration, and no neuroprotective drugs have been developed as yet.

Studies on the pathogenesis of HE and the development of neuroprotective drugs have been conducted using animal models. There are two conventional animal models of HE: one in which an isoelectric electroencephalogram (EEG) is maintained for a long period (hypoglycemic coma model) [4, 5] and the other involving euthanization or glucose administration before the isoelectric EEG (hypoglycemic non-coma model) [6–8]. In the hypoglycemic coma model, the isoelectric EEG manifesting after the BG decrease is maintained for at least 30 min to ensure that the brains are in a state of severe hypoglycemia. However, once changes in the brain due to hypoglycemia become apparent, seizures and respiratory arrest that cause additional neuronal damage are unavoidable. Model animals must therefore be treated with an anti-seizure drug and an artificial ventilator beforehand. Regarding a prolonged, markedly hypoglycemic state with an anesthetic agent and an artificial ventilator use, the pathogenesis of HE in animal models may differ from that of human HE experienced in clinical practice. However, the hypoglycemic non-coma model can also be problematic in that the various indices used by researchers as alternatives to isoelectric EEG, to indicate degrees of severity and courses, vary considerably, reflecting differences in individual responses to hypoglycemic loads. Thus, in order to elucidate the pathogenesis of HE and develop neuroprotective drugs, there is a need to establish animal models using isoelectric EEG as a quantitative index of hypoglycemic loads, without the use of anti-seizure drugs and an artificial ventilation.

Regarding the pathological conditions of HE, neuronal death in animal models is reportedly induced by oxidative stress generated after glucose administration [5, 9, 10]. This phenomenon is termed “glucose reperfusion injury”, and its severity increases with higher BG levels after BG correction [10]. In this context, we recently confirmed higher BG levels after correction in 47 consecutive patients who experienced hypoglycemic episodes (271.1 ± 128.5 mg/dL) [3]. Therefore, an animal model that experiences hypoglycemia followed by similar higher BG levels is preferred.

Studies on the development of neuroprotective drugs for HE have aimed to improve hypoenergetic conditions associated with hypoglycemia or to inhibit oxidative stress caused by glucose reperfusion injury. Among factors causing oxidative stress, 4-hydroxy-2-nonenal (4-HNE), a cytotoxic aldehyde that is a lipid oxidation product, has been intensively investigated in myocardial ischemia [11, 12]. It has been shown that 4-HNE is produced from ω-6 polyunsaturated fatty acids, such as arachidonic acid, and can cause mitochondrial disorders to potentially induce neuronal death [13]. Interestingly, *N*-(1,3-benzodioxole-5-ylmethyl)-2,6-dichlorobenzamide (Alda-1), an aldehyde dehydrogenase 2 (ALDH2) agonist that increases the activity of mitochondrial ALDH2, inhibits myocardial damage caused by 4-HNE after ischemia [12, 14]. Therefore, this raises the possibility that Alda-1 may inhibit oxidative stress caused by glucose reperfusion injury via suppression of 4-HNE levels.

Based on the aforementioned observations, this study had the following three objectives: (1) to establish a novel animal model of HE that can be maintained in an isoelectric EEG state for a short period without the use of anti-seizure drugs and an artificial ventilation, enabling the isoelectric EEG to be used as an index of HE (we termed this model “a short-term hypoglycemic coma model”), (2) to confirm whether oxidative stress and neuronal death occur in this model, and (3) to reveal whether Alda-1 exerts protective effects against neuronal death associated with glucose reperfusion injury thereby demonstrating its potential as a neuroprotective drug.

## Materials and Methods

The study protocol was approved by the Niigata University Administrative Panel on Laboratory Animal Care. All operations concerning animals were performed according to ARRIVE (Animal Research: Reporting of In Vivo Experiments) guidelines [15]. All efforts were made to minimize the number of animals used and their suffering. Alda-1 and vehicle (dimethyl sulfoxide, DMSO) were coded and administered by a blinded observer.

### EEG recordings

Male Sprague-Dawley rats (260–310 g, 8–10 weeks old, Charles River Japan, Inc.) were used. After sebum had been removed from both the shaved head and anterior neck regions by scrubbing with cotton balls soaked in alcohol, both areas were abraded with EEG abrasive gel (Skin Pure, Nihon Kohden Corporation). EEG disc electrodes (LEAD110A, BIOPAC Systems, Inc.) to which EEG disc electrode paste had been applied (Elefix, Nihon Kohden Corporation) were then attached to the forehead (near the nose) and the occiput (between the porus acusticus) of the rat, for use as EEG recording electrodes; the former served as the reference. Another disc electrode was also attached to the anterior neck to be used as the earth electrode. After the electrodes were attached, we confirmed with an alternating current (AC) resistance measuring instrument (Model 1089ES Checktrode, UFI Inc.) that the contact resistance between each electrode was 10 kΩ or less and that the variation in contact resistance at each electrode was 3 kΩ or less. An EEG amplifier (EEG100C, BIOPAC Systems, Inc.) was used for EEG amplification, and an analog-digital (A/D) converter (MP100System, BIOPAC Systems, Inc.) and waveform analysis software (AcqKnowledge, BIOPAC Systems, Inc., version 3.7.2) were used to collect and analyze the data. A bandpass of 0.1 to 100 Hz was used to filter the amplified EEG, and the sampling frequency of A/D conversion was 200 Hz.

### Short-term hypoglycemic coma model

The aforementioned rats were used. Each rat was fed a restricted diet of 20 g/day [16] for at least three consecutive days and then fasted, being allowed only water, for one day before the operation. To prevent changes in food consumption due to stress caused by an increase or decrease in the number of rats in each cage [17], the number per cage was fixed at three. The remaining amount of food was measured once a day before feeding to confirm that there were no changes in food consumption.

**Fig 1:**
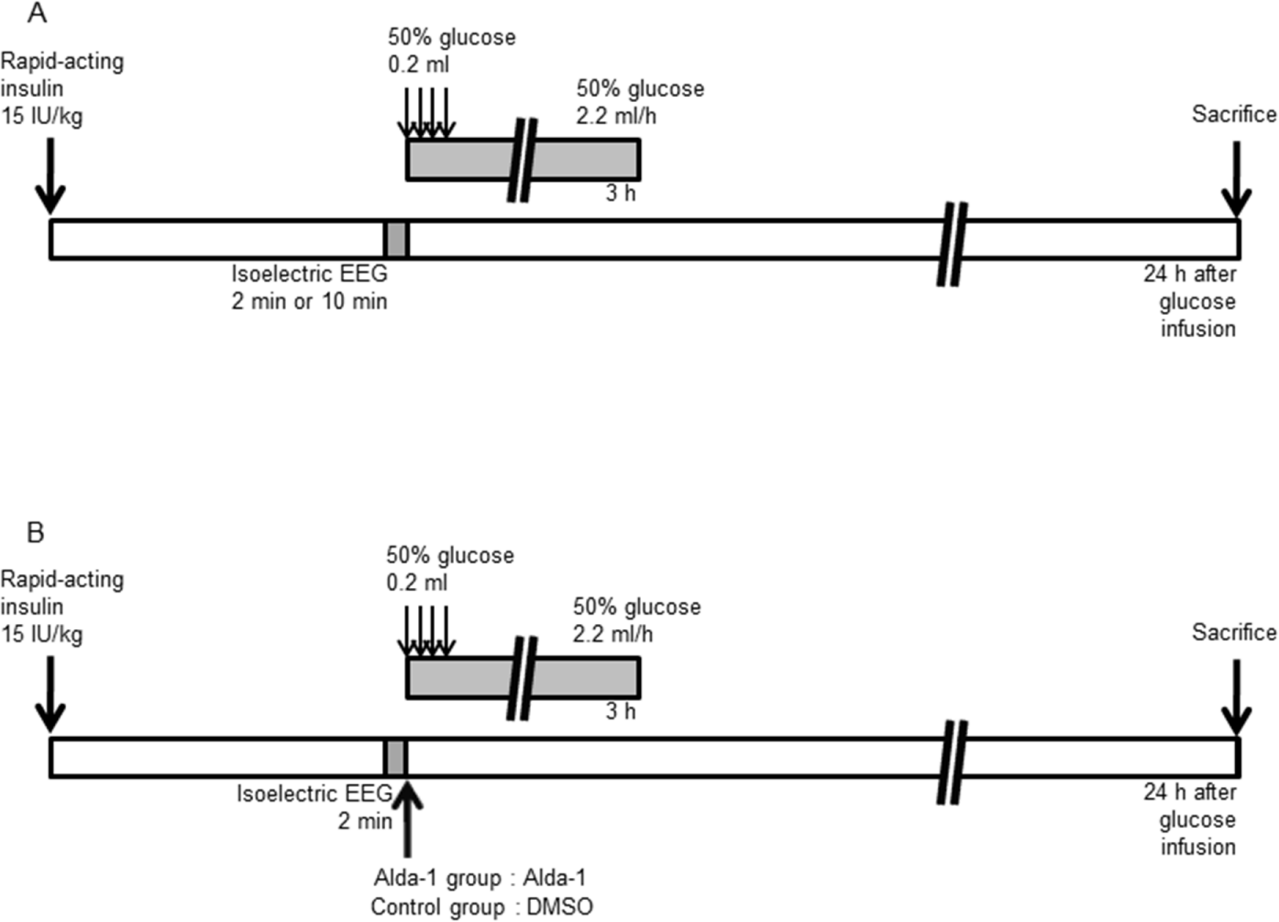
Short-term hypoglycemic coma model. (A) Protocol for hypoglycemic loading and induction of the hyperglycemic state. Rapid-acting insulin was intraperitoneally administered at a dose of 15 IU/kg. After 2 or 10 min of the presence of an isoelectric electroencephalogram (EEG), 50% glucose was intravenously injected at a dose of 0.2 mL and intravenously infused at a rate of 2.2 mL/h. Rats were sacrificed after 24 h of glucose reperfusion. (B) Alda-1 treatment protocol. After 2 min of an isoelectric EEG state, Alda-1 was administered intravenously at a dose of 8.5 mg/kg with 50% glucose (Alda-1 group) or the same dose of dimethyl sulfoxide (DMSO) was administered (control group).

Hypoglycemic loads were applied under anesthesia maintained with a mixed gas of 70% nitrous oxide and 30% oxygen with 1.0% to 1.5% halothane added while rectal temperature was monitored. The rectal temperature was maintained at 37.0 ± 0.5°C with a temperature control mat, a fan, and a heating lamp. Under direct visualization using a surgical microscope, the left inguinal region was incised to expose the left femoral vein. A polyethylene catheter (Clay Adams Intramedic polyethylene tube PE50, Becton Dickinson & Co.) filled with 1% heparinized saline was then placed in the vein and used to collect blood samples for BG measurement. A BG monitoring instrument (Ascensia BREEZE 2, Bayer Health Care) was used for BG measurement. Next, the left cervical region was incised to expose the left anterior facial vein. Another tube, as described above, was placed in the vein and used to administer glucose and experimental drugs. After a resting EEG had been recorded, rapid-acting insulin (Novolin R 100 IU/mL Injection, Novo Nordisk Pharma Co., Ltd.) was intraperitoneally administered at a dose of 15 IU/kg [5, 18], and blood samples were then collected every 15 min. If the EEG did not become isoelectric by more than 180 min after insulin administration, the experiment was discontinued. After 2 or 10 min of isoelectric EEG state, 50% glucose was intravenously injected at a dose of 0.2 mL (Fig 1A). Then, 50% glucose was intravenously infused at a rate of 2.2 mL/h and again intravenously injected at a dose of 0.2 mL three times every 5 min. Furthermore, while blood samples were collected every 15 min, an additional 50% glucose solution was administered at doses of 0.2 mL if necessary, to maintain BG levels at the target of 250 to 270 mg/dL for 3 h to create a hyperglycemic state that would cause glucose reperfusion injury in HE. BG levels after correction by glucose administration were determined by reference to our clinical trials [3]. Disc electrodes and catheters were removed 3 h after the start of glucose administration.

### Intravenous administration of an aldehyde dehydrogenase agonist

An ALDH2 agonist (Alda-1, Merck KGaA), an activator of aldehyde dehydrogenase, was dissolved in DMSO at a concentration of 15 mg/mL. At the end of the isoelectric EEG period, Alda-1 was administered to the Alda-1 group animals at a dose of 8.5 mg/kg with glucose into the left anterior facial vein, and the same dose of DMSO was administered to the control group (Fig 1B). The dose of Alda-1 was determined according to the optimal dosage described in previous reports of myocardial ischemia in rats [12, 14]. Alda-1 and DMSO were administered to randomly selected rats.

### Immunostaining for 4-HNE, a lipid oxidation product

Rats were euthanized with halothane 24 h after glucose administration. The left ventricle was perfused with 200 mL of cold saline and 100 mL of 4% paraformaldehyde (PFA) [19]. Removed whole brains were fixed in 4% PFA at 4°C for 12 h [20]. After rinsing with 0.1 M phosphate buffered saline (PBS) at 4°C overnight, brains were immersed in 15% sucrose/0.1 M PBS at 4°C overnight and then 30% sucrose/0.1 M PBS at 4°C for 24 h. Four coronal sections were collected from each animal starting 4.0 mm posterior to Bregma. They were frozen in isopentane, embedded in optimal cutting temperature (OCT) compound (Tissue-Tek, Sakura Finetek Inc.), and stored at -80°C. Brain samples were cut with a cryostat (Cryotome E Electronic Cryostat, Thermo Electron Co.) into sections of 20 μm thickness, placed on slides, and dried for 2 h. Samples were immersed in 0.3% hydrogen peroxide dissolved in absolute methanol for 30 min to inhibit peroxidase activity. After rinsing with PBS, sections were incubated with mouse monoclonal anti-4-HNE antibody (HNEJ-2, JalCA, 1:50) at 4°C overnight. The secondary antibody (#BA-2000, Vector Laboratories, Inc.) was applied at room temperature for 2.5 h. After incubation with an ABC Elite kit (Vectastain ABC, Vector Laboratories, Inc.), sections were developed in 3,3'-diaminobenzidine. Cerebral cortices (hippocampus level) from each rat were viewed using a light microscope (Eclipse E600W, Nikon Co.). The number of 4-HNE-positive cells was counted in five non-overlapping areas of 0.25 mm^2^ in both parietal and temporal cortical sections, and findings were confirmed in triplicate. Cells were counted in both hemispheres by a blinded observer.

### Assessment of neuronal death by Fluoro-Jade B

The 25-μm sections were placed on slides, dried, then immersed in 0.1 M PBS, dried at 50°C or higher for 30 min, and immersed in 80% ethanol containing 1% sodium hydroxide. Next, samples were immersed in 70% ethanol solution for 2 min and in purified water for 2 min. After immersion in 0.06% potassium permanganate solution for another 10 min, samples were rinsed with purified water for 2 min. After immersion in degenerating neuron binding fluorescent derivative staining solution (Fluoro-Jade B [FJB], Histo-Chem Inc., 0.0004%) for 20 min, a 1-min rinse with purified water was repeated three times; samples were then dried at 50°C for 15 min. Next, samples were immersed in ethanol and xylene, dehydrated, and mounted with DPX (Fluka; Sigma-Aldrich Chemie GmbH, Deisenhofen, Germany). Cerebral cortices (hippocampus level) from each rat were observed with a fluorescence microscope (excitation wavelength, 480 nm; fluorescence wavelength, 525 nm). The number of FJB-positive cells was counted in five non-overlapping areas of 0.25 mm^2^ in both parietal and temporal cortical sections, and findings were confirmed in triplicate. Cells were counted in both hemispheres by a blinded observer.

### Statistical analysis

Results are expressed as means ± standard deviation (SD). A *t*-test was used for statistical analyses. A *P* value < 0.05 was considered statistically significant.

## Results

### Changes in BG levels before and after insulin administration

**Fig 2:**
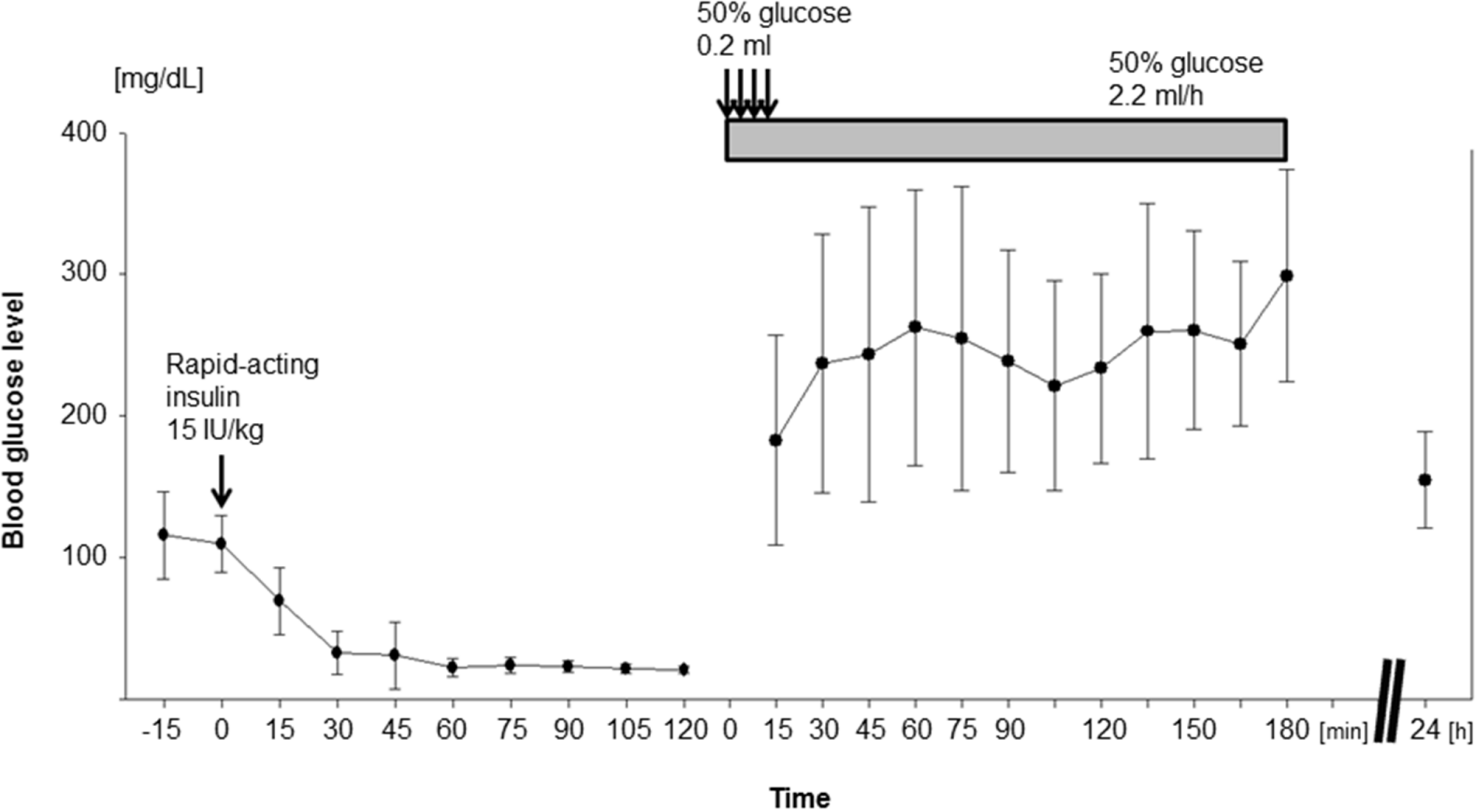
Temporal changes in blood glucose levels after administration of insulin and glucose. Blood glucose levels were measured every 15 min before and after injection of rapid-acting insulin (from -15 min to 120 min; *n* = 48) as well as after injection of 50% glucose (from 15 min to 180 min and 24 h; *n* = 9).

We first measured BG levels of rats before and after insulin administration. The mean BG level before insulin administration was 112.4 ± 26.1 mg/dL (Fig 2). After insulin administration, BG levels gradually decreased and stabilized around 20 mg/dL 60 min after insulin administration. Since their EEGs did not become isoelectric more than 180 min after insulin administration, the experiment was discontinued in 3 of 30 rats to which glucose was to be administered 2 min after an isoelectric EEG state was observed (2-min isoelectric EEG group) and 4 of 48 rats to which glucose was to be administered after 10 min of an isoelectric EEG state (10-min isoelectric EEG group). After glucose administration, BG levels immediately increased and remained in the 250 to 270 mg/dL range 30 min after the start of glucose administration and beyond.

### Changes in EEG and neurological findings

**Fig 3:**
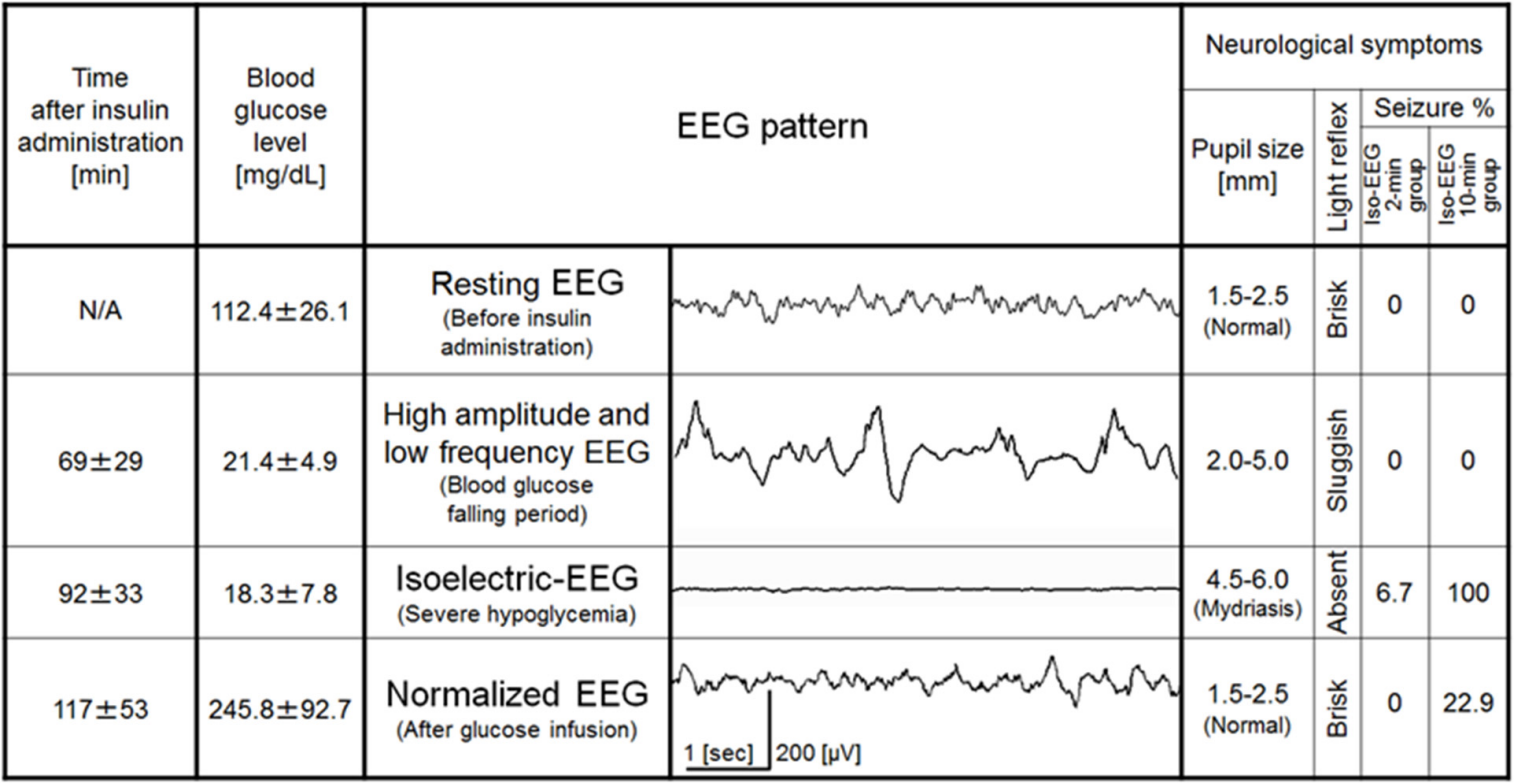
Temporal changes in electroencephalogram findings and neurological signs. EEG; Electroencephalogram, N/A; not applicable. Data are means ± SD. Since pupil diameter and light reflexes were not maintained at a constant level during each phase, these findings were not suitable for predicting precise BG levels.

We next investigated EEG findings and neurological signs associated with hypoglycemia and glucose reperfusion (Fig 3). Resting EEGs before insulin administration exhibited amplitudes of 10 to 50 μV and a frequency range of 4 to 8 Hz. There were no pupil abnormalities, and the light reflex was rapid. High-amplitude slow waves (amplitude, 100–200 μV; frequency, 0.1–4 Hz) occurred 69 ± 29 min after insulin administration. At this time, BG levels were 21.4 ± 4.9 mg/dL, pupil diameter was increased, and the light reflex was slow. Mean EEG amplitude and frequency then both gradually decreased, and the EEG became isoelectric 92 ± 33 min after insulin administration. At this time, BG levels were 18.3 ± 7.8 mg/dL, pupils were dilated, and the light reflex disappeared. When glucose was administered after isoelectric EEG, the appearance of the EEG was similar to that at rest (recovering EEG). Following glucose administration, BG levels were 245.8 ± 92.7 mg/dL, dilatation of the pupils disappeared, and the light reflex was rapid. Since pupil diameter and light reflexes were not maintained at a constant level during each phase, these findings were not suitable for predicting precise BG levels. Frequencies of seizure and subsequent death in the isoelectric EEG 2-min group were 6.7% (2/30) and 16.7% (5/30), respectively. By contrast, these were 100% (48/48) and 87.5% (42/48), respectively, in the 10-min isoelectric EEG group.

### Assessment of 4-HNE-positive cells in the short-term hypoglycemic coma model

**Fig 4:**
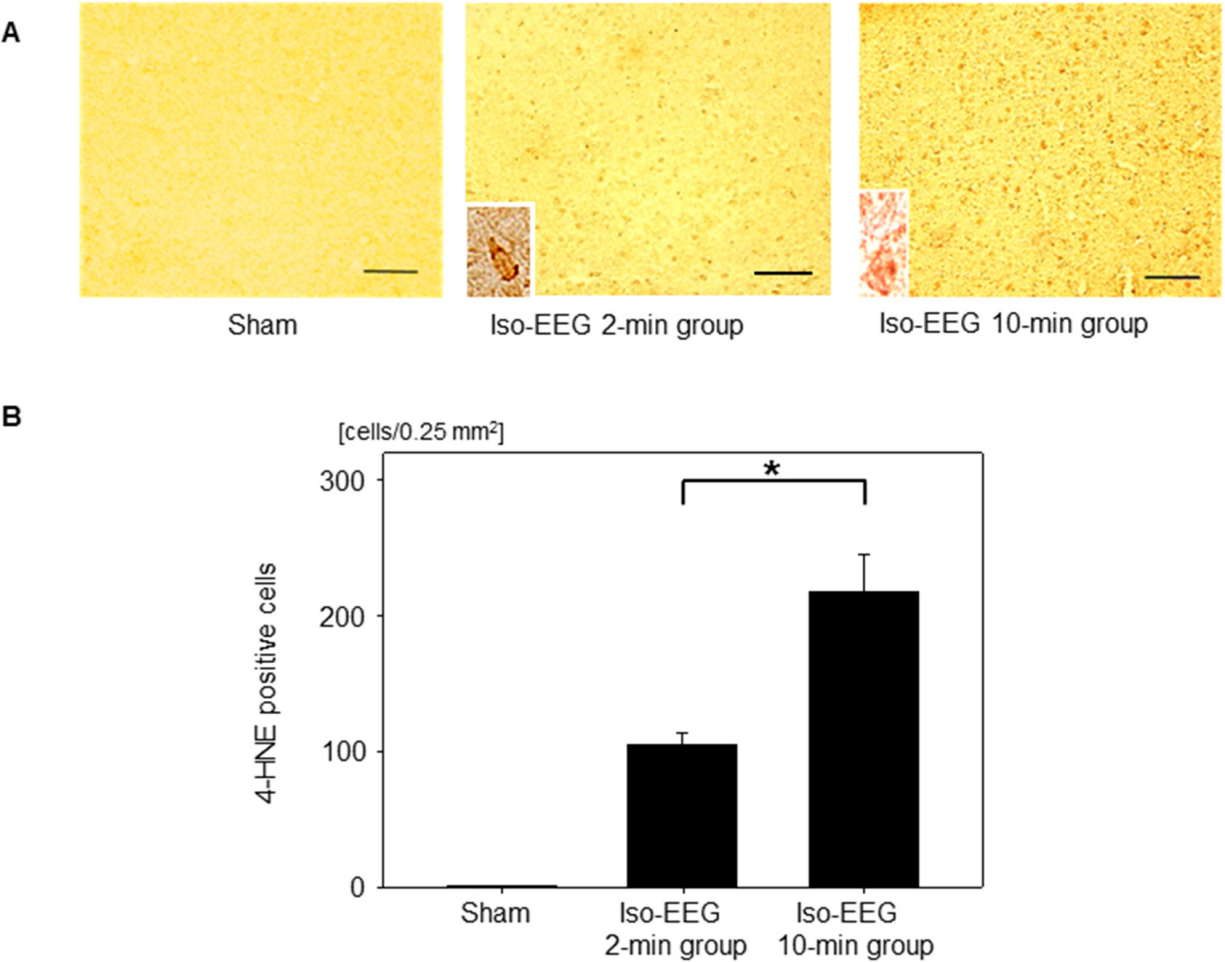
Distribution of 4-HNE-positive cells in the short-term hypoglycemic coma model. (A) Representative images of 4-HNE-positive cells in the sham group, the 2-min isoelectric EEG (iso-EEG) group, and the 10-min isoelectric EEG group. Higher magnification views of 4-HNE-positive cells are shown in the small frames. Scale bar: 100 μm. (B) The number of 4-HNE-positive cells in the sham group, the 2-min isoelectric EEG group, and the 10-min isoelectric EEG group. Data are means ± SD (*n* = 15 fields). *P < 0.001.

The number of 4-HNE-positive cells was compared among the sham group (to which insulin was not administered), the 2-min isoelectric EEG group, and the 10-min isoelectric EEG group (Fig 4). There were no significant differences in body weight, food consumption, or BG levels before or after the experiment among the three groups. In the sham group, no 4-HNE-positive cells were detected in any of the brain regions examined. In the 2-min isoelectric EEG group, 4-HNE-positive cells were detected in the frontal to parietal-temporal cortex and in the hippocampus. The number of 4-HNE-positive cells in the hippocampus was less than that in the cerebral cortex. The frequency of 4-HNE-positive cells in the parietal-temporal cortex at the level of the hippocampus was significantly higher in the 10-min isoelectric EEG group than in the 2-min isoelectric EEG group (217.9 ± 27.1 cells/0.25 mm^2^ vs. 104.8 ± 8.9 cells/0.25 mm^2^; *P* < 0.001).

### Assessment of FJB-positive cells in the short-term hypoglycemic coma model

The frequency of hypoglycemia-induced neuronal death, evaluated by FJB staining of the parietal-temporal cortex at the level of the hippocampus, was significantly higher in the 10-min isoelectric EEG without glucose reperfusion group than in the 2-min isoelectric EEG without glucose reperfusion group (109.9 ± 45.4 cells/0.25 mm^2^ vs. 53.3 ± 15.9 cells/0.25 mm^2^; P = 0.009, S1 Fig).

**Fig 5:**
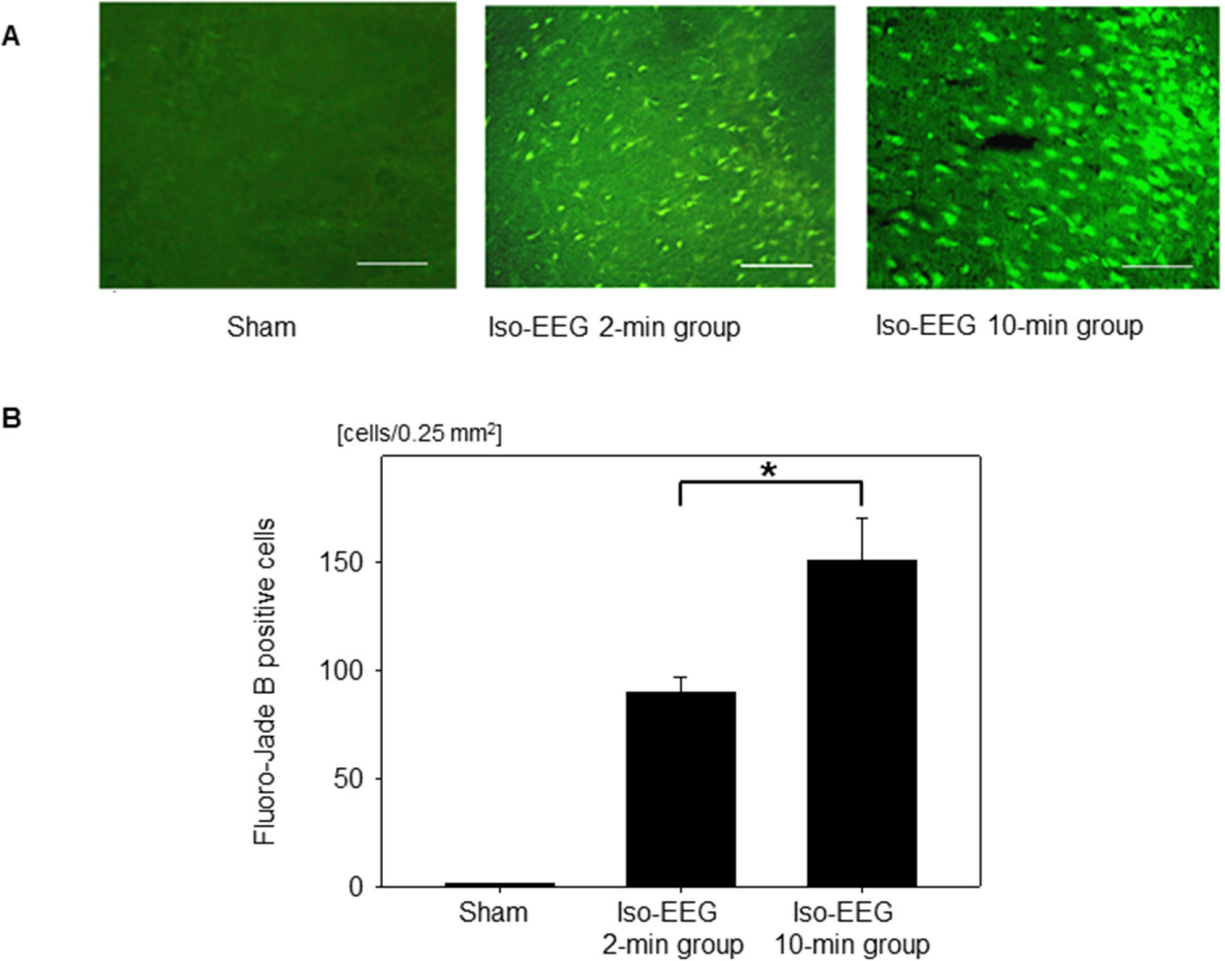
Fluoro-Jade B-positive cells in the short-term hypoglycemic coma model. (A) Representative images of Fluoro-Jade B-positive cells in the sham group, the 2-min isoelectric EEG (iso-EEG) group, and the 10-min isoelectric EEG group. Scale bar: 100 μm. (B) The number of Fluoro-Jade B-positive cells in the sham group, the 2-min isoelectric EEG group, and the 10-min isoelectric EEG group. Data are means ± SD (*n* = 15 fields). *P < 0.001.

Next, the number of FJB-positive cells was compared among the sham, 2-min isoelectric EEG, and 10-min isoelectric EEG groups (Fig 5). In the sham group, there were no FJB-positive cells in any of the brain regions examined. In the 2-min isoelectric EEG group, FJB-positive cells were observed in the frontal to parietal-temporal cortex and hippocampus. The number of FJB-positive cells in the hippocampus was less than that in the cerebral cortex. The frequency of FJB-positive cells in the parietal-temporal cortex at the level of the hippocampus was significantly higher in the 10-min isoelectric EEG group than in the 2-min isoelectric EEG group (150.7 ± 19.8 cells/0.25 mm2 vs. 89.9 ± 6.8 cells/0.25 mm2; P < 0.001).

### Effects of Alda-1 on 4-HNE-positive cells

**Fig 6:**
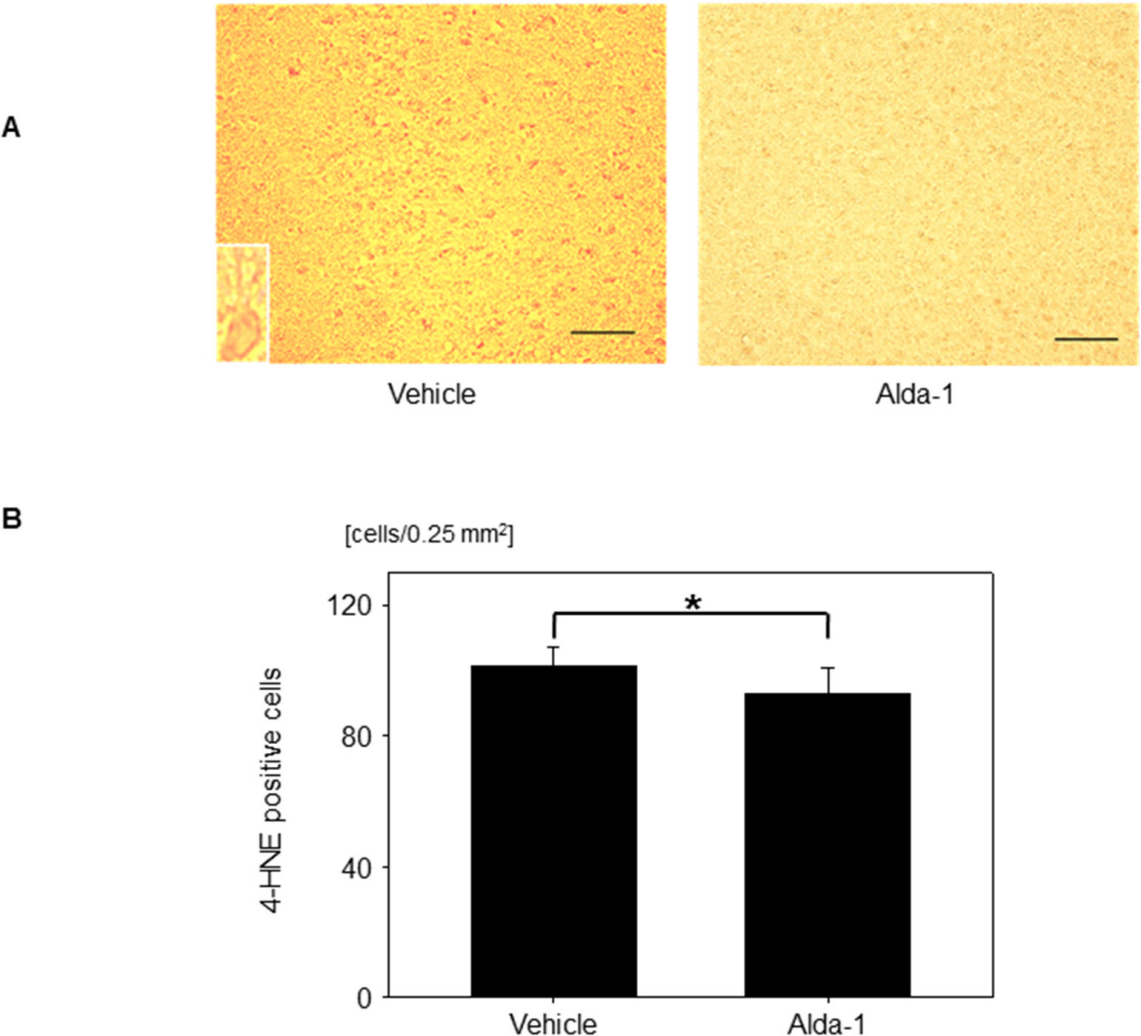
Effects of Alda-1 on 4-HNE-positive cells. (A) Representative images of 4-HNE-positive cells treated with vehicle (dimethyl sulfoxide; DMSO) or Alda-1. A higher magnification view of a 4-HNE-positive cell is shown in the small frame. Scale bar: 100 μm. (B) Number of 4-HNE-positive cells in groups treated with vehicle (DMSO) or Alda-1. Data are means ± SD (*n* = 15 fields). *P = 0.002.

To examine the effects of Alda-1 on glucose reperfusion-associated 4-HNE expression, the number of 4-HNE-positive cells was examined in rats to which Alda-1 had been administered after 2 min of isoelectric EEG (Alda-1 group) and in rats to which DMSO, a solvent, had been administered (control group). There were no significant differences in body weight, food consumption, or BG levels between the two groups. The number of 4-HNE-positive cells was significantly lower in the Alda-1 group than in the control group (101.5 ± 5.6 vs. 92.9 ± 8.1 cells/0.25 mm^2^; *P* = 0.002) (Fig 6). We did not examine the effect of Alda-1 in the 10-min isoelectric EEG group because a large number of rats developed seizures before administration of glucose and Alda-1.

### Effects of Alda-1 on FJB-positive cells

**Fig 7:**
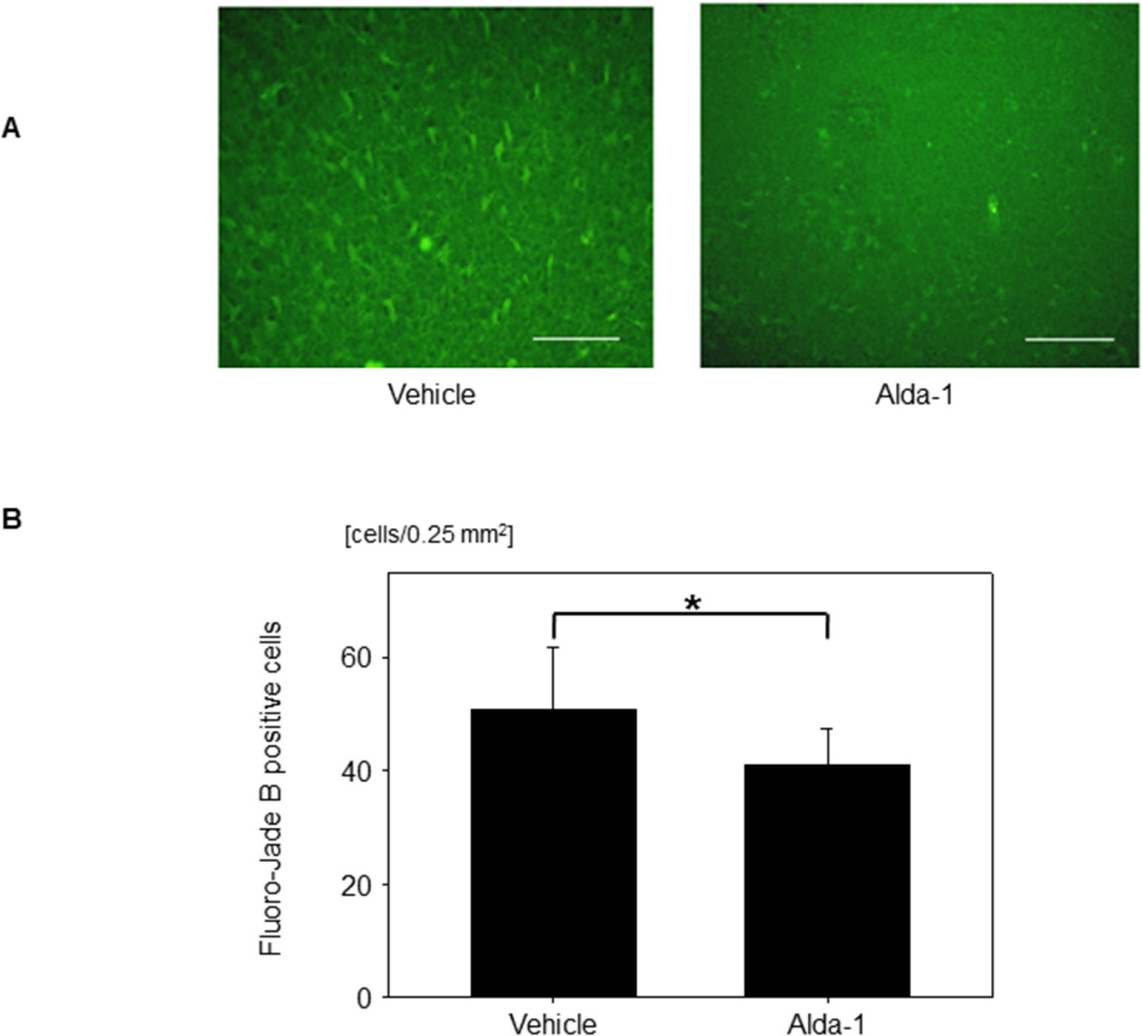
Effects of Alda-1 on Fluoro-Jade B-positive cells. (A) Representative images of Fluoro-Jade B-positive cells treated with vehicle (DMSO) or Alda-1. Scale bar: 100 μm. (B) Number of Fluoro-Jade B-positive cells in groups treated with vehicle (DMSO) or Alda-1. Data are means ± SD (*n* = 15 fields). *P = 0.020.

Finally, to ascertain whether Alda-1 exerts protective effects against glucose reperfusion-associated neuronal death, the number of FJB-positive cells was compared in the Alda-1 and control groups. Notably, the number of FJB-positive cells was significantly lower in the Alda-1 group than in the control group (50.9 ± 11.1 vs. 41.1 ± 6.3 cells/0.25 mm^2^; *P* = 0.020) (Fig 7).

## Discussion

In this study, we first attempted to develop a short-term hypoglycemic coma model that could be maintained in an isoelectric EEG state for a short period. The isoelectric EEG period was reduced from 30 min, which is the isoelectric period used in a conventional hypoglycemic coma model, to 2 min. We confirmed that seizures did not occur in this condition. The advantages of this model are as follows. (i) The degree of severity induced by the hypoglycemic load is relatively uniform, while measurements of the light reflex and pupil diameter lack objectivity and quantitativity. (ii) BG levels during the hyperglycemic state are determined on the basis of actual human clinical data [3]. (iii) This model does not require the use of anti-seizure drugs and an artificial ventilator. (iv) The severity of oxidative stress and neuronal death are quantifiable by measuring 4-HNE- and FJB-positive cells, respectively.

Next, we investigated the characteristics of glucose reperfusion injury using this model, and noted the following observations. (i) We found that the appearance of 4-HNE-positive cells in the brain even in the 2-min isoelectric EEG group. This suggests that the conditions present in the 2-min isoelectric EEG group are enough to cause glucose reperfusion injury. Since oxidative stress is not caused by hypoglycemic loads alone [7, 10], this raises the possibility that hyperglycemia associated with glucose administration causes glucose reperfusion injury. (ii) We observed fewer 4-HNE-positive cells in the 2-min isoelectric EEG group than in the 10-min isoelectric EEG group, although BG levels during the hyperglycemic state were similar. This finding suggests that the severity of glucose reperfusion injury may be influenced by the degree of severity of the hypoglycemic load-induced neuronal damage. However, to our knowledge, no reports have determined the effect of the severity of the hypoglycemic load on glucose reperfusion injury. Future studies need to address this issue. (iii) We observed fewer 4-HNE-positive cells in the hippocampus than in the cerebral cortex. Previous studies demonstrated similar findings in the non-coma model [7, 8], although the mechanism underlying the difference in 4-HNE production in different brain regions remains unknown.

Finally, we showed that administration of Alda-1 inhibits both the production of 4-HNE and neuronal death associated with glucose reperfusion injury, similar to previous reports in which Alda-1 inhibits myocardial damage caused by 4-HNE after myocardial ischemia [12, 14]. Previous studies show that treatments devised to attenuate oxidative stress associated with glucose reperfusion injury may be therapeutic candidates, including therapeutic hypothermia [19], administration of nitric oxide synthase inhibitors [21], and nicotine adenine dinucleotide phosphate oxidase inhibitors [10]. In addition, Alda-1 may be a therapeutic candidate. Activation of ALDH2 by Alda-1 increases detoxification of reactive aldehydes, such as 4-HNE, a cytotoxic aldehyde that accumulates during glucose reperfusion injury as a by-product of ROS-induced lipid peroxidation [14]. Enhanced ALDH2 activity reduces 4-HNE protein adduct formation and increases cell survival. We suggest that activation of the ALDH2 pathway could be a molecular target for HE treatment, and that Alda-1 is a potentially neuroprotective agent that exerts a beneficial effect on neurons when intravenously administered simultaneously with glucose.

This study has a number of limitations. First, with regard to the FJB-positive cells in the 10-min isoelectric EEG group, the seizures associated with a prolonged hypoglycemic state may affect the results. Second, we did not investigate the effect of other neuroprotective drugs that inhibit oxidative stress on neuronal death. Future studies are required to determine the most suitable drug for treatment of glucose reperfusion injury.

In conclusion, we established a short-term hypoglycemic coma model. In this model, we confirmed both the appearance of 4-HNE-positive cells and neuronal death associated with glucose reperfusion injury. Furthermore, with this model we demonstrated that Alda-1 inhibits 4-HNE production and exerts a protective effect against neuronal death. Thus, Alda-1 may be a candidate neuroprotective agent for cases involving HE.

## Supporting Information

S1 Fig Hypoglycemia-induced neuronal death evaluated by Fluoro-Jade B staining. (A) Representative images of Fluoro-Jade B-positive cells in the sham group, the 2-min isoelectric EEG (iso-EEG) without glucose reperfusion group, and the 10-min isoelectric EEG without glucose reperfusion group. Scale bar: 100 μm. (B) The number of Fluoro-Jade B-positive cells in the sham group, the 2-min isoelectric EEG without glucose reperfusion group, and the 10-min isoelectric EEG without glucose reperfusion group. Data are means ± SD (*n* = 15 fields). *P = 0.009.
(TIF)
