# Supplementary figures and images for: Effects of Alda-1, an Aldehyde Dehydrogenase-2 Agonist, on Hypoglycemic Neuronal Death
Source: PLoS One. 2015 Jun 17;10(6):e0128844. doi: 10.1371/journal.pone.0128844 (PMC4471358; doi:10.1371/journal.pone.0128844)

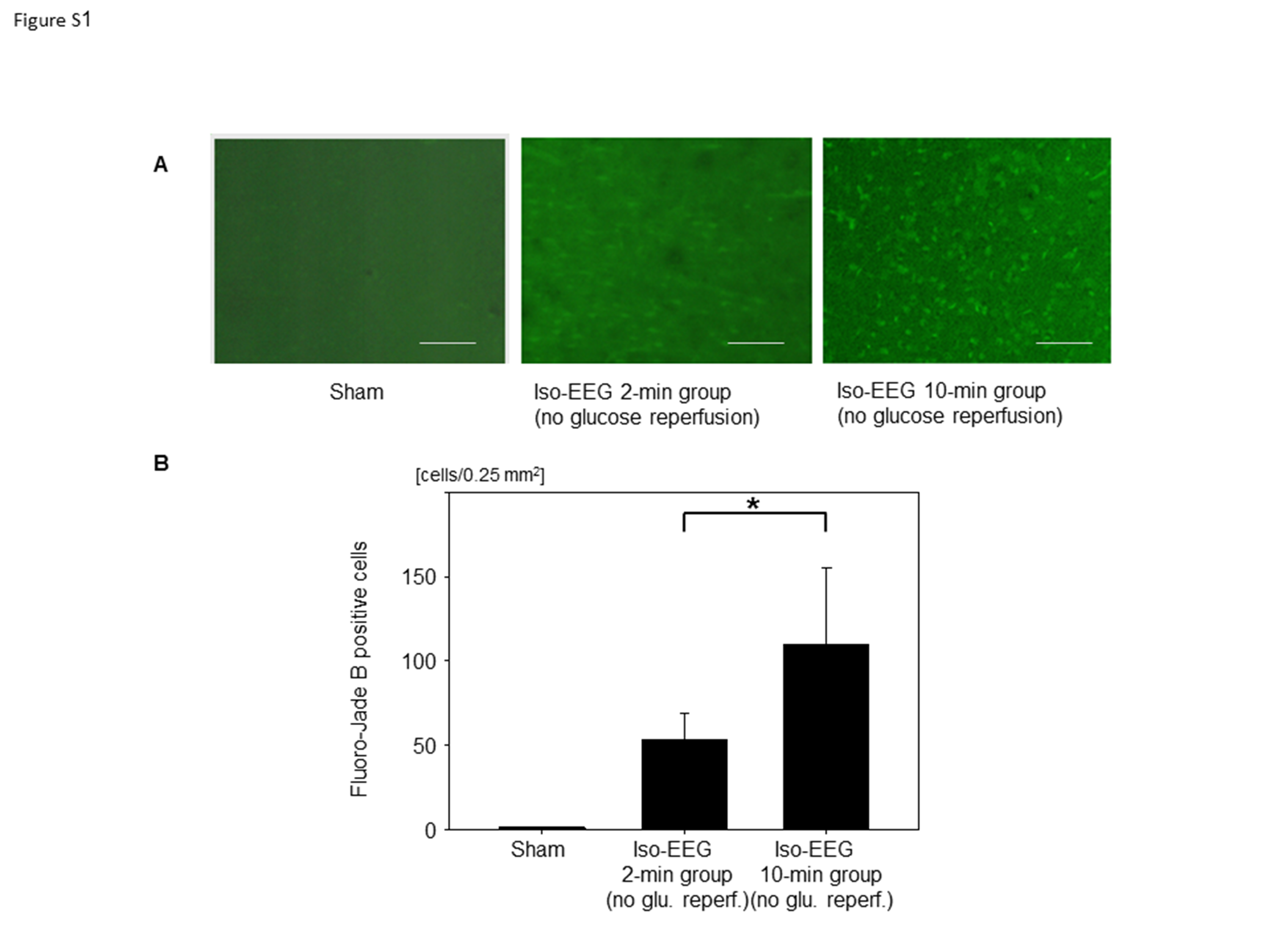

Supplement: S1 Fig — (A) Representative images of Fluoro-Jade B-positive cells in the sham group, the 2-min isoelectric EEG (iso-EEG) without glucose reperfusion group, and the 10-min isoelectric EEG without glucose reperfusion group. Scale bar: 100 μm. (B) The number of Fluoro-Jade B-positive cells in the sham group, the 2-min isoelectric EEG without glucose reperfusion group, and the 10-min isoelectric EEG without glucose reperfusion group. Data are means ± SD (n = 15 fields). *P = 0.009. (TIF) [file pone.0128844.s001.TIF]
